# Thermal Water Reduces the Inflammatory Process Induced by the SARS-CoV-2 Spike Protein in Human Airway Epithelial Cells In Vitro

**DOI:** 10.3390/biomedicines12122917

**Published:** 2024-12-21

**Authors:** Anna Scanu, Maria Chiara Maccarone, Fabrizio Caldara, Gianluca Regazzo, Roberto Luisetto, Stefano Masiero

**Affiliations:** 1 Rehabilitation Unit, Department of Neuroscience-DNS, University of Padova, Via Giustiniani 2, 35128 Padova, Italy; mariachiara.maccarone93@gmail.com (M.C.M.); stef.masiero@unipd.it (S.M.); 2 Pietro d’Abano Thermal Studies Center, Via Jappelli 5, 35031 Abano Terme, Italy; fabrizio.caldara@centrostuditermali.org; 3 Physical Medicine and Rehabilitation School, University of Padova, Via Giustiniani 2, 35128 Padova, Italy; gianlucareg@gmail.com; 4 Department of Surgical Oncological and Gastroenterological Sciences-DISCOG, University of Padova, Via Giustiniani 2, 35128 Padova, Italy; roberto.luisetto@unipd.it

**Keywords:** SARS-CoV-2, COVID-19, spike protein, balneotherapy, inflammation, thermal water

## Abstract

**Background**: Although treatments using thermal water have yielded beneficial effects in respiratory tract infections, the effects of thermal water under experimental conditions similar to those triggered by SARS-CoV-2 have yet to be evaluated. This study aimed to assess whether thermal water could interfere with the interaction between SARS-CoV-2 and host cells and influence inflammatory factors. **Methods**: Human nasal epithelial primary cells (HNEpCs) were stimulated with SARS-CoV-2 spike protein in the presence or absence of thermal water or tap water. Cell viability, cytokine concentration, ACE2 and TMPRSS2 levels, and ACE2 activity were determined in the cell cultures. **Results**: Exposure of HNEpCs to spike protein increased IL-6, IL-8, and IL-1β production, with decreased production observed in the presence of thermal water at an optimal dose. Treatment of cells with tap water did not affect cytokine release in unstimulated or spike-stimulated cells. Spike-protein-stimulated HNEpCs showed reduced levels of ACE2, which were partially restored only in the presence of thermal water. Spike protein did not affect the TMPRSS2 levels of the cell lysates. Stimulation with spike protein induced an increase in the concentration of both receptors in the supernatants, while treatment with thermal water reduced TMPRSS2 levels in both the cells and supernatants. Stimulation with spike protein increased ACE2 activity, which was reduced with thermal water. **Conclusions**: This study shows the regulatory effects of mineral-rich thermal water on spike-protein-induced pro-inflammatory cytokine production and the amount and activity of receptors mainly involved in viral entry, suggesting a potential use of this treatment as a support therapy for SARS-CoV-2 infection of the upper respiratory tract.

## 1. Introduction

Balneotherapy is an effective, well-tolerated, complementary approach commonly used in the treatment of different conditions, especially rheumatic and dermatological disorders [1,2,3,4]. This therapy includes all treatments that involve immersion or contact with mineral water derived directly from a natural spring. Therefore, this definition also includes physical activities performed in these types of waters and the application of hyperthermal mud baths directly from mineral springs [5]. Although precise mechanisms of action for balneotherapy remain elusive, several factors have been suggested to contribute to its therapeutic effects.

In recent years, several studies have highlighted that the beneficial effects of balneotherapy are due to its immunomodulatory and anti-inflammatory properties. In particular, preclinical and clinical studies reported that treatment with thermal mineral waters can regulate the production of inflammatory mediators, thereby reducing the levels of C-reactive protein and the release of pro-inflammatory cytokines such as TNF-α, IL-1β, and IL-6 and promoting the release of anti-inflammatory molecules such as IGF-1. Furthermore, balneotherapy influences the activity of matrix metalloproteinases (MMPs), thus reducing inflammatory tissue damage. Balneotherapy can also inhibit the production of prostaglandins, helping to reduce pain associated with inflammation. In addition to the main inflammatory mediators, balneotherapy has also been observed to modulate the effector populations of the immune system, particularly by increasing T-reg subsets such as the CD4+CD25+FOXP3 T subpopulation and T-helper cells and by decreasing the number of circulating antigen-4 cytotoxic lymphocytes and natural killer lymphocytes [6,7,8]. Finally, current scientific evidence shows that by promoting relaxation and reducing stress, the spa environment and the therapeutic effects of mineral-rich baths can positively impact the regulation of circulating cortisol levels. A recent meta-analysis indicated a short-term reduction in cortisol levels induced by balneotherapy in healthy subjects experiencing high levels of stress. Conversely, temporary increases in cortisol concentrations were observed in elderly patients with rheumatic conditions undergoing balneotherapy, reducing inflammatory mediators and improving pain and functional quality of life [9].

In addition to traditional bathing, treatments that use mineral-rich thermal water can also incorporate inhalation of the water’s vapors or nasal irrigation. Inhaled thermal water vapor particles with a diameter of around 20 μm come into contact with the epithelia of the upper respiratory tract, which is an important physical barrier in humans and contains cells of the innate and adaptive immune system. This type of application has shown curative effects in various respiratory diseases such as allergic rhinitis, asthma, chronic obstructive pulmonary disease (COPD) [10], and infections [11] by reducing symptoms, inflammation, oxidant and antioxidant imbalance, and cellular influx; increasing fluidity while normalizing the overproduction of mucus; and improving pulmonary and nasal function and quality of life [12,13]. The use of thermal mineral-rich water is widely accepted as an effective therapeutic approach for upper respiratory tract infections. The inhalation of thermal water under such conditions has led to a significant reduction in the frequency, duration, and severity of infectious episodes, potentially due to its antiviral and antibacterial effects. This hypothesis is supported by the observed decrease in viral replication in airway epithelial cell cultures and lower counts of nasal bacteria, along with the inhibition of bacterial biofilm production in patients after thermal water administration [11]. Nasal irrigation, i.e., rinsing the nasal cavities with thermal water inserted through the nostrils, has also shown some positive effects in treating and preventing recurrent upper respiratory tract infections, especially among pediatric patients [14,15,16]. Given this encouraging evidence, the spa environment may have the potential to serve as a suitable out-of-hospital setting for respiratory rehabilitation in post-coronavirus disease 2019 (COVID-19) contexts [17,18]. However, although data in the literature have reported beneficial activities against almost all manifestations of respiratory disorders, the exact mechanisms remain unknown.

The outbreak of the COVID-19 infection caused a significant reduction in the activities of many spas around the world, including the Euganean Thermal Basin in Italy. This reduction was not only related to compliance with measures established by the authorities to contain the spread of COVID-19 but also associated with the cancelation of a considerable number of bookings, even when the facilities were authorized to reopen. However, currently, there is no evidence suggesting that COVID-19 can spread to people through the water used in pools [19], and specific interactions between water and virus macromolecules have not been thoroughly evaluated.

The waters of the Euganean Thermal Basin belong to the category of salt–bromine–iodine waters according to the classification of Marotta and Sica [20,21]. These waters are highly mineralized, and spring at temperatures between 65 and 87 °C and have shown anti-inflammatory activities [22]. Recently, it was reported that hydrogen sulfide (H2S), a natural compound found in sulfurous waters, reduces the in vitro expression of transmembrane protease serine protease-2 (TMPRSS2) in human upper and lower airway epithelial cells. These findings suggest that exposing respiratory epithelial cells to H2S sources may impede the entry of SARS-CoV-2 into airway epithelial cells, potentially preventing the virus from spreading to the lower respiratory tract [23]. The interactions between viral structural proteins and host cells are known to play key roles in the onset of the disease. Indeed, the SARS-CoV-2 spike glycoprotein S starts the infection process by binding to human membrane receptors ACE2 (angiotensin-converting enzyme-2), allowing the virus to fuse with the cell membrane and enter the cell [24]. Virus entry into the cell is facilitated by host proteases such as TMPRSS-2 [25,26]. However, the effects of thermal water in experimental conditions that mimic the physiopathological context of SARS-CoV-2 have never been evaluated, nor have the interactions between salt–bromine–iodine waters, host cells, and the SARS-CoV-2 virus.

Therefore, the objectives of this study were to evaluate whether salt–bromine–iodine water could interfere with the interactions between SARS-CoV-2 and host cells and the resulting influence on some inflammatory aspects. To reproduce conditions similar to those triggered by SARS-CoV-2, human airway epithelial cells, which are the first cellular targets of coronavirus infection, were stimulated in vitro with spike protein. Our results indicate that mineral-rich thermal water reduces the production of pro-inflammatory cytokines induced by SARS-CoV-2 spike protein in vitro. These effects may be due to the regulation of the level and activity of the receptors involved in virus entry.

## 2. Materials and Methods

### 2.1. Thermal Water Collection

Thermal water at a temperature of 78 °C was collected in sterile glass bottles directly from the source in a spa of the Euganean Thermal Area located in Abano Terme and cooled to 37 °C before performing the experiments. The chemical–physical properties of the Abano Terme (Padua, Italy) salsobromoiodic thermal environment were previously reported [27]. The experiments were carried out on the same day the thermal water was collected to maintain the water’s chemical–physical properties.

### 2.2. Cell Culture

Human nasal epithelial primary cells (HNEpCs) were obtained from PromoCell (PromoCell, Heidelberg, Germany) and cultured in an airway epithelial cell growth medium (PromoCell, Heidelberg, Germany) containing bovine pituitary extract (0.004 mL/mL), L-epidermal growth factor (recombinant human) (10 ng/mL), insulin (recombinant human) (5 µg/mL), hydrocortisone (0.5 µg/mL), epinephrine (0.5 µg/mL), triiodo-L-thyronine (6.7 ng/mL), transferrin (recombinant human) (10 µg/mL), and retinoic acid (0.1 ng/mL) according to the manufacturer’s recommendations. This serum-free medium is optimized for the in vitro cultivation of epithelial cells from large air passages.

Cells were maintained in a 5% CO_2_ air-humidified atmosphere at 37 °C. Subculture and seeding were performed using a Detach Kit (PromoCell, Heidelberg, Germany) according to the manufacturer’s protocol. Briefly, cells were first washed with Hepes BSS (HEPES buffered balanced salt solution) and then incubated in the presence of Trypsin/EDTA solution until detachment. The trypsinization reaction was stopped by adding Trypsin Neutralization Solution. Cellular morphological alterations were monitored with microscope observations.

### 2.3. MTT Assay

Cell viability and proliferation were measured using a colorimetric MTT (3-(4,5-dimethylthiazol-2-yl)-2,5-diphenyltetrazolium bromide) mitochondrial activity assay (Sigma-Aldrich, St. Louis, MO, USA). Briefly, cells were seeded in a 96-well plate at 37 °C and exposed to varying percentages of thermal water in a culture medium. After 24 h, the cells were incubated with the MTT solution for 4 h. Then, dimethyl sulfoxide was added to dissolve the resulting formazan crystals, and the absorbance value was measured at 570 nm. Cell viability (%) was reported as a percentage relative to the untreated cells.

### 2.4. Cell Stimulation

HNEpCs were stimulated for 24 h with the spike protein of the SARS-CoV-2 Omicron variant (B.1.1.529) (1 µg/mL) (Twin Helix, Milano, Italy) [28] in an airway epithelial cell growth medium in the presence or absence of thermal water. Alternatively, tap water was used instead of thermal water. The properties of the tap water are available on the website of the water distribution company for the municipality of Padua [29]. Cells treated with the medium only or the medium containing thermal water or tap water served as control groups. At the end of the experiments, cells and supernatants were collected for subsequent analysis.

### 2.5. Cytokine Determination

Culture supernatants were tested with an enzyme-linked immunosorbent assay (ELISA) for the production of IL-6, IL-8, and IL-1β (Biolegend, San Diego, CA, USA).

### 2.6. ACE2 and TMPRSS2 Determination

Extracellular ACE2 and TMPRSS2 concentrations were evaluated in culture supernatant by ELISA (Raybiotech, Norcross, GA, USA). The total amounts of ACE2 and TMPRSS2 present in the cells were determined in lysates obtained after three freeze–thaw cycles and resuspended in phosphate-buffered saline (PBS) using the same methods (Fine Biotech Co., Wuhan, China) [30].

### 2.7. ACE2 Activity Assay

ACE2 activity in HNEpCs was measured using a commercially available assay kit that measures the ability of an active ACE2 to cleave a synthetic 7-Methoxycoumarin-4-acetic acid (MCA)-based peptide substrate to release a free fluorophore (Sigma-Aldrich, St. Louis, MO, USA). Briefly, cells were lysed using a specific buffer supplied within the kit, and the total protein content per sample was determined using the Bradford assay. The total protein content of the culture supernatants was detected by the same method. Then, cell lysates or culture supernatants were plated in a 96-well plate in addition to the appropriate standards and controls. Next, an ACE2 substrate was added, and fluorescence was measured at a 320 nm excitation and 420 nm emission wavelength. The ACE2 activity for each sample was calculated using the following formula:

ACE2 activity = B × D/ΔT × P

where B is the released MCA (cleaved product of ACE2 substrate) in the sample based on the standard curve slope, ΔT is the reaction time (in min), P is the sample used (in mg), and D is the sample dilution factor.

### 2.8. Statistical Analysis

Results are presented as the mean ± SD of 4 independent experiments. Statistical differences were determined using a nonparametric Mann–Whitney test; *p* < 0.05 was considered significant.

## 3. Results

### 3.1. Effect of Thermal Water on Spike-Protein-Induced Cytokine Production

**Figure 1:**
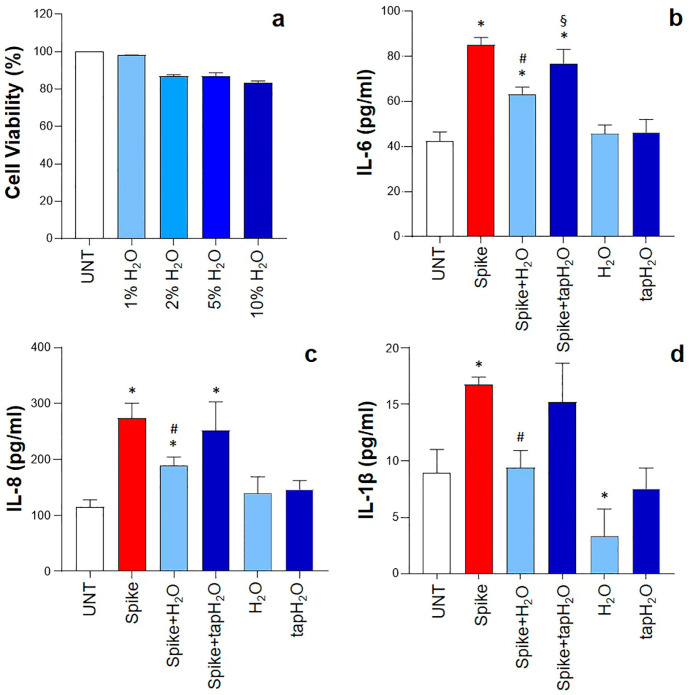
Effect of thermal water on cytokine production in human nasal epithelial primary cells (HNEpCs). The cells were treated with increasing percentages of thermal water (H_2_O). Cell viability was then evaluated after 24 h (**a**). Cells were stimulated (or not) for 24 h with 1 µg/mL SARS-CoV-2 spike protein in the presence or absence of 1% thermal water or tap water. Culture supernatants were analyzed for the production of IL-6 (**b**), IL-8 (**c**), and IL-1β (**d**). Results are presented as the mean ± SD of four separate experiments. * *p* < 0.05 vs. untreated (UNT) cells; # *p* < 0.05 vs. spike-protein-stimulated cells; § *p* < 0.05 vs. spike-protein-stimulated cells treated with thermal water.

Thermal water can induce osmotic stress within in vitro cultures. Thus, in order to identify the optimal experimental conditions, HNEpCs were treated with increasing percentages of thermal water, and cell activity was examined. The results demonstrated that cell viability was reduced to less than 90% when the percentage of thermal water in the culture medium was between 2% and 10%. However, cell viability only slightly decreased (cell viability > 98%) when the water content did not exceed 1% (Figure 1a). Therefore, in subsequent experiments, HNEpCs were treated with an optimal dose of 1% thermal water.

In the absence of a stimulus, HNEpCs released low levels of IL-6, IL-8, and IL-1β in the culture supernatants. These levels significantly increased when the cells were exposed to spike protein. As shown in Figure 1b–d, the levels of all evaluated cytokines in spike-stimulated cells were double those of untreated cells. Treatment of epithelial cells with thermal water caused a significant inhibition of cytokine secretion induced by the spike protein. Indeed, in the presence of thermal water, IL-6, IL-8, and IL-1β concentrations were reduced by 26%, 31%, and 44%, respectively. Furthermore, IL-1β levels were found to be lower in the supernatants of cells treated only with thermal water than under basal conditions. Interestingly, treating cells with tap water (1%) did not affect cytokine production via either unstimulated or spike-stimulated cells.

### 3.2. Effect of Thermal Water on ACE2 and TMPRSS2 Receptor Levels

**Figure 2:**
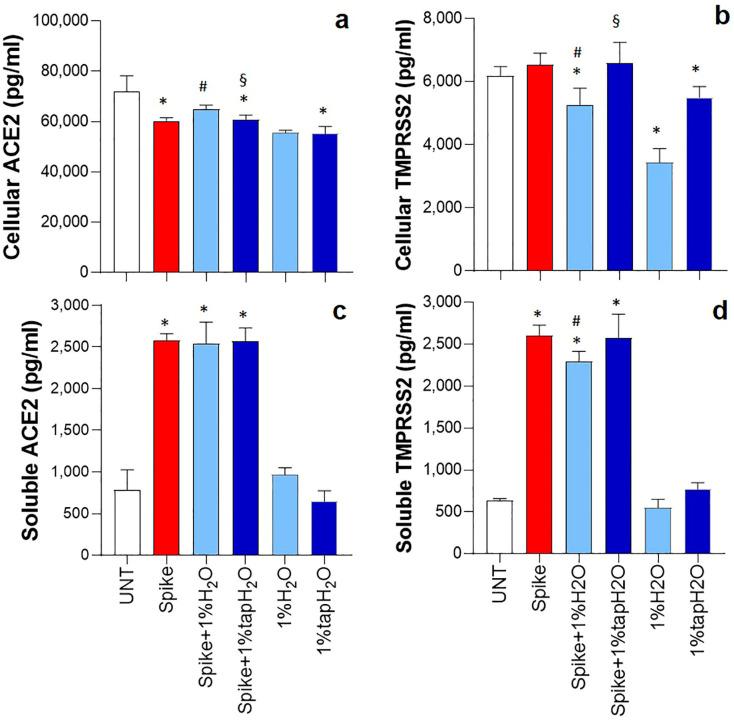
Effect of thermal water on the levels of SARS-CoV-2 spike protein receptors. Human nasal epithelial primary cells (HNEpCs) were stimulated (or not stimulated) for 24 h with 1 µg/mL SARS-CoV-2 spike protein in the presence or absence of 1% thermal water (H_2_O) or tap water. The concentrations of ACE2 and TMPRSS2 were determined in cell lysates (**a**,**b**). In addition, ACE2 and TMPRSS2 levels were determined in culture supernatants (**c**,**d**). Results are presented as the mean ± SD of four separate experiments. * *p* < 0.05 vs. untreated (UNT) cells; # *p* < 0.05 vs. spike-protein-stimulated cells; § *p* < 0.05 vs. spike-protein-stimulated cells treated with thermal water.

To determine whether ACE2 and TMPRSS2 might play a role in the effect of thermal water on cytokine release, the levels of the two receptors were assessed. As shown in Figure 2a, exposure of HNEpCs to spike protein significantly reduced the levels of ACE2 (16% compared to untreated cells), which were partially restored (8% compared to spike-stimulated cells) only in the presence of thermal water. Otherwise, the spike protein did not significantly affect TMPRSS2 levels in the cell lysates. However, thermal water significantly reduced the concentration of this receptor in both spike-stimulated and unstimulated cell lysates (Figure 2b). Indeed, thermal water decreased the levels of TMPRSS2 by 20% and 44%, respectively, in spike-activated and unstimulated cells. The presence of tap water in the culture supernatant did not affect the levels of TMPRSS2 in spike-stimulated cells.

Because ACE2 sheds from the membrane and increased concentrations of soluble ACE2 (sACE2) are found in patients with COVID-19 [31,32], levels of sACE2 were determined in the culture supernatants. The concentration of sACE2 increased significantly by three-fold after stimulation with spike protein and was similar by either thermal water or tap water treatment (Figure 2c). Exposing HNEpCs to thermal or tap water also did not alter the levels of ACE2 observed under basal conditions. Interestingly, evaluation of the supernatants also highlighted an increase in the concentration of TMPRSS2 induced by the spike protein. Conversely, this concentration was reduced after treatment with thermal water (Figure 2d).

### 3.3. Effect of Thermal Water on ACE2 Activity

**Figure 3:**
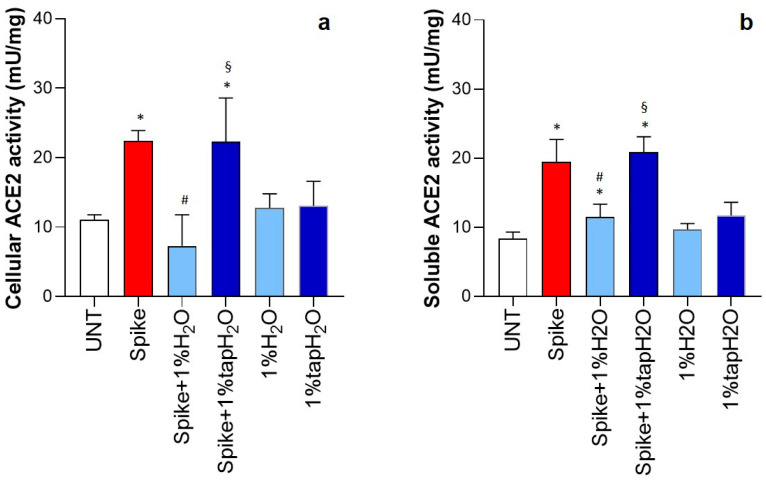
Effect of thermal water on ACE2 activity in human nasal epithelial primary cell (HNEpC) cultures. HNEpCs were stimulated (or not) with 1 µg/mL SARS-CoV-2 spike protein in the presence or absence of 1% thermal water (H_2_O) or tap water. ACE2 activity was evaluated after 24 h in cells (**a**) and supernatants (**b**). Results are presented as the mean ± SD of four separate experiments. * *p* < 0.05 vs. untreated (UNT) cells; # *p* < 0.05 vs. spike-protein-stimulated cells; § *p* < 0.05 vs. spike-protein-stimulated cells treated with thermal water.

To further characterize how thermal water exerts its anti-inflammatory effects, ACE2 activity was evaluated in cells and supernatants. As shown in Figure 3a, ACE2 activity in cells was significantly increased (two-fold) in spike-stimulated HNEpC compared to that in the controls but remained at basal levels in the presence of thermal water. After treatment with tap water, the ACE2 activity of spike-activated cells was significantly higher than that of both unstimulated and protein-stimulated cells in the presence of thermal water. In the absence of a stimulus, ACE2 activity was not affected by either thermal water or tap water. Similar results were obtained for ACE2 activity in the supernatants (Figure 3b).

## 4. Discussion

This study indicates that treatment with an optimal dose of mineral-rich thermal water can reduce pro-inflammatory cytokine release induced by SARS-CoV-2 spike protein in airway epithelial cells. The in vitro model used suggests that the mechanism of action involved the regulation of the number of ACE2 and TMPRSS2 receptors present in the cells and ACE2 activity.

ACE2 is not only a critical regulator of cardiovascular, renal, gastrointestinal, and respiratory physiology and pathology but also plays a pivotal role in driving the susceptibility and severity of COVID-19. In particular, it has been shown that tissue-bound ACE2 and sACE2 levels may be an important factor in predisposing susceptibility to various COVID-19-related pathologies. Binding of the spike glycoprotein to the ACE2 receptor is crucial for the entry of the virus into host cells. Thus, the inhibition of this initial step is important to prevent or limit infection. Several papers have reported that individual SARS-CoV-2 proteins, particularly the spike protein itself, can exert pathologic effects [33]. For example, various membrane-associated SARS-CoV-2 proteins have been demonstrated to induce an immune response, evidenced by an increase in pro-inflammatory cytokine expression in both THP-1 and human peripheral blood mononuclear cells. Such proteins were also found to elicit inflammation in BALB/c mice after intranasal administration [34]. Furthermore, both in vitro and in vivo experiments demonstrated that SARS-CoV-2 spike (S) and envelope (E) surface proteins drive airway epithelial cells to induce inflammation independent of viral infection and replication [35]. IL-1 was suggested to play an important role in this type of inflammation. Indeed, in vitro models of hypercoagulation and inflammation showed that spike protein induces platelet activation through engagement with CD42b and contributes to the activation of the Nlrp3 inflammasome and IL-1β production in stem cells [28,36]. More recently, it was observed that the SARS-CoV-2 spike protein receptor binding domain promotes the release of IL-6 and IL-8 in human bronchial epithelia [37]. Here, in addition to IL-1β, the SARS-CoV-2 spike protein alone was able to induce the production of IL-6 and IL-8 in airway epithelial cells. These results support the premise that spike protein can also exert a direct effect on cells and cause a signaling response. In our experiments, cytokine release was inhibited in the presence of thermal water, thus confirming the anti-inflammatory effects of this water. Indeed, several papers have reported the capacity of thermal water to reduce the production of pro-inflammatory cytokines, including IL-1β, IL-6, and IL-8, and counteract other inflammatory processes in different in vitro disease models and clinical trials [6,7,8].

Most studies evaluating the effects of balneotherapy were conducted on musculoskeletal and skin disorders, demonstrating the regulation of key factors involved in disease pathogenesis and progression, as well as in symptom improvement. However, the benefits of treatment with mineral-rich thermal waters have also been observed in respiratory infections, for which it was proposed that immunomodulatory activity could be accompanied by antimicrobial action [12,13]. In this context, sulfurous thermal water has yielded therapeutic effects, and its administration via inhalation was reported to decrease the amount of bacteria and bacterial biofilm production in patients with recurrent respiratory infections, such as chronic bronchitis and rhinosinusitis [14,38,39]. This observation was associated with the direct and indirect local toxicity of hydrogen sulfide, which can lead to eradication of the bacterial film [40,41]. In addition, the use of salt–bromo–iodine waters can be useful in the management of upper airway infections. Nasal nebulization with this type of water was found to be effective in the treatment of children with upper respiratory tract infections and otitis media with effusion, showing a significant improvement in symptoms and functional recovery. In both cases, the authors speculated that these effects may be due to a significant improvement in mucociliary clearance time (MCT), an activity that generally prevents organic, inorganic, bacterial, or viral particles from entering the organism [42,43]. However, previous works evaluating the effect of salt–bromo–iodine water only reported impacts on the symptoms of infection, while the mechanism of action remains completely unexplored.

In this study, salt–bromo–iodine water from the Euganean basin seemed to exert its effects by modulating the number and activity of receptors that play a key role in SARS-CoV-2 infection. In our experiments, stimulation with spike protein induced a reduction in the amount of ACE2 present in cells, possibly due to the increased release of this receptor, a decrease in ACE2 mRNA expression and induction of cell-bound ACE2 degradation, as demonstrated by previous studies [44,45,46]. Furthermore, in agreement with previous investigations conducted in vitro and in human plasma, we observed increased ACE2 activity after spike protein stimulation in both cells and supernatants [47,48]. After treatment with mineral-rich thermal water, changes in the concentration of cell lysates and the activity of ACE2 receptors induced by spike protein stimulation tended to be reduced, suggesting that thermal mineral-rich water might interfere in the viral protein–cellular receptor interactions. This interference could be due to the presence of essential minerals, such as iodine, which was shown to alter the structure of antigenic glycoproteins found on the surfaces of some viruses, thus modifying the sites of interaction between the virus and host receptors and retaining antimicrobial activity without cytotoxic effects against mammalian cells [49,50]. However, we observed that the spike protein induced increased sACE2 protein levels, which remained similar in the presence of thermal water too. Because it increased cell-bound ACE2 but did not decrease soluble ACE2, thermal water may act directly on host cells by inducing the production of new cell-bound protein receptors without affecting the mechanisms involved in shedding of ACE2.

Interestingly, we observed that stimulation with spike protein induced an increase in TMPRSS2 levels in the culture supernatants, suggesting that the receptor may be released from cells after activation by the protein. Notably, although soluble forms of TMPRSS2 have been described [51,52], to our knowledge, this is the first study evaluating the release of TMPRSS2 from cells following activation by SARS-CoV-2 spike protein. Treatment with thermal water also induced a modulation of the TMPRSS2 receptor, whose levels were reduced in both cell lysates and supernatants in the presence of the spike protein, as well as in lysates of unstimulated cells. This result suggests that mineral-rich thermal water may contain molecules that induce the down-regulation of this receptor. In this context, Pozzi et al. obtained interesting results in a recent in vitro study evaluating the effects of H2S donors on TMPRSS2 receptor expression in human airway epithelial cells. In this study, H2S was observed to selectively down-regulate TMPRSS2 expression in airway epithelial cells without significantly affecting ACE2 expression [23].

This study has some limitations. The first limitation relates to the use of spike protein and not the whole virus for HNEpC stimulation. Although spike protein plays a key role in the entry of SARS-CoV-2 into infected cells and the associated pro-inflammatory stimulus, other structural proteins of SARS-CoV-2 such as those of the envelope or nucleocapsid contribute to the infection itself and the respiratory syndromes caused by the virus itself. Another important limitation is related to the fact that an animal model was not used in the experiment involving inhalation of salsobromoiodine water vapors or thermal bathing of the entire model organism exposed to SARS-CoV-2. However, the limited generalizability of available animal models in the study of SARS-CoV-2 and their poor performance, due mainly to differences in their pulmonary physiology and immunology compared to those of humans, should be considered. Furthermore, laboratory rodent strains require the transgenic expression of human ACE-2 to allow the effective entry of the virus into respiratory tract cells. In addition, our in vitro model does not differentiate mild from severe disease. Therefore, future studies should also consider this parameter. Finally, in this study, experiments were carried out by using a classic (i.e., submerged) in vitro model, in which thermal water interacts with the HNEpC monolayer via the culture medium. This setting effectively mimics procedures that involve immersion or nasal washing but is less representative of practices such as the aerosolization or inhalation of nebulized water or thermal vapors. However, the model used here is a useful tool to improve knowledge of the cellular effects of thermal water treatment and allowed us to directly evaluate the action of this water on the activity of soluble ACE2. Furthermore, even though air–liquid interface inhalation models are increasingly used for respiratory system research, no in vitro methods have yet been validated or standardized to sufficient levels [53]. Only recently a novel bionebulizer approach was developed to study the effects of mineral water [54]. Future studies should attempt to replicate these administration routes for thermal water and specifically consider the route of contact with the respiratory tract.

## 5. Conclusions

In conclusion, this study demonstrates that mineral-rich thermal water reduces the production of pro-inflammatory cytokines induced by SARS-CoV-2 spike protein in vitro and that the regulation of the key receptors involved in virus entry might be the mechanism responsible for this effect. Although further studies are needed to better elucidate the exact mechanisms, our results suggest that treatment with mineral-rich thermal water may help mucosal defense by minimizing contact between the mucosa and viral particles, thus limiting virus binding and internalization into host cells. Consequently, this study not only strengthens the potential for using mineral-rich thermal water treatment as support therapy for upper respiratory infections but also provides valuable information indicating that such treatment does not facilitate spread of the virus.

## Data Availability

Data are contained within the article.

## References

[1] Maccarone M.C., Magro G., Albertin C., Barbetta G., Barone S., Castaldelli C., Manica P., Marcoli S., Mediati M., Minuto D. (2023). Short-time effects of spa rehabilitation on pain, mood and quality of life among patients with degenerative or post-surgery musculoskeletal disorders. Int. J. Biometeorol..

[2] Cozzi F., Ciprian L., Carrara M., Galozzi P., Zanatta E., Scanu A., Sfriso P., Punzi L. (2018). Balneotherapy in chronic inflammatory rheumatic diseases-a narrative review. Int. J. Biometeorol..

[3] Huang A., Seité S., Adar T. (2018). The use of balneotherapy in dermatology. Clin. Dermatol..

[4] Cacciapuoti S., Luciano M.A., Megna M., Annunziata M.C., Napolitano M., Patruno C., Scala E., Colicchio R., Pagliuca C., Salvatore P. (2020). The role of thermal water in chronic skin diseases management: A review of the literature. J. Clin. Med..

[5] Gutenbrunner C., Bender T., Cantista P., Karagülle Z. (2010). A proposal for a worldwide definition of health resort medicine, balneology, medical hydrology and climatology. Int. J. Biometeorol..

[6] Maccarone M.C., Magro G., Solimene U., Scanu A., Masiero S. (2021). From in vitro research to real life studies: An extensive narrative review of the effects of balneotherapy on human immune respons. Sport. Sci. Health.

[7] Cheleschi S., Tenti S., Seccafico I., Gálvez I., Fioravanti A., Ortega E. (2022). Balneotherapy year in review 2021: Focus on the mechanisms of action of balneotherapy in rheumatic diseases. Environ. Sci. Pollut. Res. Int..

[8] Scanu A., Tognolo L., Maccarone M.C., Masiero S. (2021). Immunological events, emerging pharmaceutical treatments and therapeutic potential of balneotherapy on osteoarthritis. Front. Pharmacol..

[9] Antonelli M., Fasano F., Veronesi L., Donelli D., Vitale M., Pasquarella C. (2024). Balneotherapy and cortisol levels: An updated systematic review and meta-analysis. Int. J. Biometeorol..

[10] Contoli M., Gnesini G., Forini G., Marku B., Pauletti A., Padovani A., Casolari P., Taurino L., Ferraro A., Chicca M. (2013). Reducing agents decrease the oxidative burst and improve clinical outcomes in COPD patients: A randomised controlled trial on the effects of sulphurous thermal water inhalation. Sci. World J..

[11] Viegas J., Esteves A.F., Cardoso E.M., Arosa F.A., Vitale M., Taborda-Barata L. (2019). Biological effects of thermal water-associated hydrogen sulfide on human airways and associated immune cells: Implications for respiratory diseases. Front. Public Health.

[12] Zajac D. (2021). Inhalations with thermal waters in respiratory diseases. J. Ethnopharmacol..

[13] Fesyun A.D., Solimene U., Grishechkina I.A., Lobanov A.A., Andronov S.V., Popov A.I., Yakovlev M.Y., Ivanova E., Sanina N.P., Reverchuk I.V. (2023). Mineral water inhalations for bronchial asthma: A meta-analysis. Eur. J. Transl. Myol..

[14] Varricchio A., Giuliano M., Capasso M., Del Gaizo D., Ascione E., De Lucia A., Avvisati F., Capuano F., De Rosa G., Di Mauro F. (2013). Salso-sulphide thermal water in the prevention of recurrent respiratory infections in children. Int. J. Immunopathol. Pharmacol..

[15] Franz L., Manica P., Claudatus J., Frigo A.C., Marioni G., Staffieri A. (2021). Sulfurous-arsenical-ferruginous thermal water nasal inhalation and irrigation in children with recurrent upper respiratory tract infections: Clinical outcomes and predictive factors. Am. J. Otolaryngol..

[16] Casale M., Moffa A., Cassano M., Carinci F., Lopez M.A., Trecca E.M.C., Torretta S., Rinaldi V., Pignataro L. (2018). Saline nasal irrigations for chronic rhinosinusitis: From everyday practice to evidence-based medicine. An update. Int. J. Immunopathol. Pharmacol..

[17] Maccarone M.C., Masiero S. (2021). Spa therapy interventions for post respiratory rehabilitation in COVID-19 subjects: Does the review of recent evidence suggest a role?. Environ. Sci. Pollut. Res..

[18] Maccarone M.C., Coraci D., Regazzo G., Sarandria N., Scanu A., Masiero S. (2023). Evolution of musculoskeletal symptoms in Long COVID: Have world health systems been prepared to deal with the Long COVID syndrome?. Joint Bone Spine.

[19] Girón-Navarro R., Linares-Hernández I., Castillo-Suárez L.A. (2021). The impact of coronavirus SARS-CoV-2 (COVID-19) in water: Potential risks. Environ. Sci. Pollut. Res. Int..

[20] Marotta D., Sica C. (1933). Composizione e classificazione delle acque minerali italiane. Ann. Chim. Appl..

[21] Messini M., Guadagnino G., Messini M. (1951). Acque sulfuree. Trattato di Idroclimatologia Clinica.

[22] Pellegrini M., Fanin D., Nowicki Y., Guarnieri G., Bordin A., Faggian D., Lebani M., Saetta M., Maestrelli P. (2005). Effect of inhalation of thermal water on airway inflammation in chronic obstructive pulmonary disease. Respir. Med..

[23] Pozzi G., Masselli E., Gobbi G., Mirandola P., Taborda-Barata L., Ampollini L., Carbognani P., Micheloni C., Corazza F., Galli D. (2021). Hydrogen sulfide inhibits TMPRSS2 in human airway epithelial cells: Implications for SARS-CoV-2 infection. Biomedicines.

[24] Zhang H., Penninger J.M., Li Y., Zhong N., Slutsky A.S. (2020). Angiotensin-converting enzyme 2 (ACE2) as a SARS-CoV-2 receptor: Molecular mechanisms and potential therapeutic target. Intensive Care Med..

[25] Hoffmann M., Kleine-Weber H., Schroeder S., Krüger N., Herrler T., Erichsen S., Schiergens T.S., Herrler G., Wu N.H., Nitsche A. (2020). SARS-CoV-2 cell entry depends on ACE2 and TMPRSS2 and is blocked by a clinically proven protease inhibitor. Cell.

[26] Koch J., Uckeley Z.M., Doldan P., Stanifer M., Boulant S., Lozach P.Y. (2021). TMPRSS2 expression dictates the entry route used by SARS-CoV-2 to infect host cells. EMBO J..

[27] Masiero S., Vittadini F., Ferroni C., Bosco A., Serra R., Frigo A.C., Frizziero A. (2018). The role of thermal balneotherapy in the treatment of obese patient with knee osteoarthritis. Int. J. Biometeorol..

[28] Li T., Yang Y., Li Y., Wang Z., Ma F., Luo R., Xu X., Zhou G., Wang J., Niu J. (2022). Platelets mediate inflammatory monocyte activation by SARS-CoV-2 spike protein. J. Clin. Investig..

[29] Water Quality in Padua. https://www.acegasapsamga.it/servizi/acqua/qualita-acqua-potabile/qualita-acqua-di-padova.

[30] Scanu A., Lorenzin M., Luisetto R., Galozzi P., Ortolan A., Oliviero F., Doria A., Ramonda R. (2022). Identification in synovial fluid of a new potential pathogenic player in arthropathies. Exp. Biol. Med..

[31] Wissing S.I., Obeid R., Rädle-Hurst T., Rohrer T., Herr C., Schöpe J., Geisel J., Bals R., Abdul-Khaliq H. (2022). Concentrations of soluble angiotensin converting enzyme 2 (sACE2) in children and adults with and without COVID-19. J. Clin. Med..

[32] Wang J., Zhao H., An Y. (2022). ACE2 Shedding and the Role in COVID-19. Front. Cell Infect. Microbiol..

[33] Theoharides T.C. (2022). Could SARS-CoV-2 spike protein be responsible for long-COVID syndrome?. Mol. Neurobiol..

[34] Haystead T., Lee E., Cho K., Gullickson G., Hughes P., Krafsur G., Freeze R., Scarneo S. (2023). Investigation of SARS-CoV-2 individual proteins reveals the in vitro and in vivo immunogenicity of membrane protein. Sci. Rep..

[35] Anand G., Perry A.M., Cummings C.L., St Raymond E., Clemens R.A., Steed A.L. (2021). Surface proteins of SARS-CoV-2 drive airway epithelial cells to induce IFN-dependent inflammation. J. Immunol..

[36] Ratajczak M.Z., Bujko K., Ciechanowicz A., Sielatycka K., Cymer M., Marlicz W., Kucia M. (2021). SARS-CoV-2 entry receptor ACE2 is expressed on very small CD45-precursors of hematopoietic and endothelial cells and in response to virus spike protein activates the Nlrp3 inflammasome. Stem Cell Rev. Rep..

[37] Zhang R.G., Liu X.J., Guo Y.L., Chen C.L. (2024). SARS-CoV-2 spike protein receptor binding domain promotes IL-6 and IL-8 release via ATP/P2Y2 and ERK1/2 signaling pathways in human bronchial epithelia. Mol. Immunol..

[38] Bellometti S., Bertocco E., Galzigna L. (1998). Changes in the Intrabronchial Microflora of Patients with Chronic Bronchitis After Inhaling Mineral Water. Vopr. Kurortol. Fizioter. Lech. Fiz. Kult..

[39] Cantone E., Marino A., Ferranti I., Castagna G., Maione N., Dirubbo V., Lengo M. (2014). Nasal cytological assessment after crenotherapy in the treatment of chronic rhinosinusitis in the elderly. Int. J. Immunopathol. Pharmacol..

[40] Albertini M.C., Dacha M., Teodori L., Conti M.E. (2007). Drinking mineral waters: Biochemical effects and health implications—The state-of-the-art. Int. J. Environ. Health.

[41] Salami A., Dellepiane M., Crippa B., Mora F., Guastini L., Jankowska B., Mora R. (2008). Sulphurous water inhalations in the prophylaxis of recurrent upper respiratory tract infections. Int. J. Pediatr. Otorhinolaryngol..

[42] La Mantia I., Andaloro C. (2018). Effects of salso-bromo-iodine thermal water in children suffering from otitis media with effusion: A randomized controlled pilot study. Clin. Ther..

[43] La Mantia I., Ciprandi G., Varricchio A., Cupido F., Andaloro C. (2018). Salso-bromo-iodine thermal water: A nonpharmacological alternative treatment for postnasal drip-related cough in children with upper respiratory tract infections. J. Biol. Regul. Homeost. Agents.

[44] Gao X., Zhang S., Gou J., Wen Y., Fan L., Zhou J., Zhou G., Xu G., Zhang Z. (2022). Spike-mediated ACE2 down-regulation was involved in the pathogenesis of SARS-CoV-2 infection. J. Infect..

[45] Sui Y., Li J., Venzon D.J., Berzofsky J.A. (2021). SARS-CoV-2 spike protein suppresses ACE2 and type I interferon expression in primary cells from macaque lung bronchoalveolar lavage. Front. Immunol..

[46] Lu Y., Zhu Q., Fox D.M., Gao C., Stanley S.A., Luo K. (2022). SARS-CoV-2 down-regulates ACE2 through lysosomal degradation. Mol. Biol. Cell.

[47] Lu J., Sun P.D. (2020). High affinity binding of SARS-CoV-2 spike protein enhances ACE2 carboxypeptidase activity. J. Biol. Chem..

[48] Patel S.K., Juno J.A., Lee W.S., Wragg K.M., Hogarth P.M., Kent S.J., Burrell L.M. (2021). Plasma ACE2 activity is persistently elevated following SARS-CoV-2 infection: Implications for COVID-19 pathogenesis and consequences. Eur. Respir. J..

[49] Gottardi W., Block S. (1991). Iodine and iodine compounds. Disinfection, Sterilization, and Preservation.

[50] Sriwilaijaroen N., Wilairat P., Hiramatsu H., Takahashi T., Suzuki T., Ito M., Ito Y., Tashiro M., Suzuki Y. (2009). Mechanisms of the action of povidone-iodine against human and avian influenza A viruses: Its effects on hemagglutination and sialidase activities. Virol. J..

[51] Lee J.H., Lee C.E., Yoo Y., Shin E., An J., Park S.Y., Song W.-J., Kwon H.-S., Cho Y.S., Moon H.-B. (2022). Soluble ACE2 and TMPRSS2 levels in the serum of asthmatic patients. J. Korean Med. Sci..

[52] Afar D.E., Vivanco I., Hubert R.S., Kuo J., Chen E., Saffran D.C., Raitano A.B., Jakobovits A. (2001). Catalytic cleavage of the androgen-regulated TMPRSS2 protease results in its secretion by prostate and prostate cancer epithelia. Cancer Res..

[53] Lacroix G., Koch W., Ritter D., Gutleb A.C., Larsen S.T., Loret T., Zanetti F., Constant S., Chortarea S., Rothen-Rutishauser B. (2018). Air-liquid interface *in vitro* models for respiratory toxicology research: Consensus workshop and recommendations. Appl. In Vitro Toxicol..

[54] Viegas J., Cardoso E.M., Bonneau L., Esteves A.F., Ferreira C.L., Alves G., Santos-Silva A.J., Vitale M., Arosa F.A., Taborda-Barata L. (2024). A novel bionebulizer approach to study the effects of natural mineral water on a 3D in vitro nasal model from allergic rhinitis patients. Biomedicines.

